# Loneliness and social isolation as risk factors for coronary heart disease and stroke: systematic review and meta-analysis of longitudinal observational studies

**DOI:** 10.1136/heartjnl-2015-308790

**Published:** 2016-04-18

**Authors:** Nicole K Valtorta, Mona Kanaan, Simon Gilbody, Sara Ronzi, Barbara Hanratty

**Affiliations:** 1 Department of Health Sciences, University of York, Heslington, York, UK; 2 Department of Health Sciences, University of York, Heslington, York, UK; 3 Department of Health Sciences, University of York, Heslington, York, UK; 4 Department of Public Health and Society, Whelan Building, Brownlow Hill, Liverpool, UK; 5 Institute of Health and Society/Newcastle University Institute for Ageing, Biomedical Research Building, Campus for Ageing and Vitality, Newcastle University, Newcastle upon Tyne, UK

## Abstract

**Background:**

The influence of social relationships on morbidity is widely accepted, but the size of the risk to cardiovascular health is unclear.

**Objective:**

We undertook a systematic review and meta-analysis to investigate the association between loneliness or social isolation and incident coronary heart disease (CHD) and stroke.

**Methods:**

Sixteen electronic databases were systematically searched for longitudinal studies set in high-income countries and published up until May 2015. Two independent reviewers screened studies for inclusion and extracted data. We assessed quality using a component approach and pooled data for analysis using random effects models.

**Results:**

Of the 35 925 records retrieved, 23 papers met inclusion criteria for the narrative review. They reported data from 16 longitudinal datasets, for a total of 4628 CHD and 3002 stroke events recorded over follow-up periods ranging from 3 to 21 years. Reports of 11 CHD studies and 8 stroke studies provided data suitable for meta-analysis. Poor social relationships were associated with a 29% increase in risk of incident CHD (pooled relative risk: 1.29, 95% CI 1.04 to 1.59) and a 32% increase in risk of stroke (pooled relative risk: 1.32, 95% CI 1.04 to 1.68). Subgroup analyses did not identify any differences by gender.

**Conclusions:**

Our findings suggest that deficiencies in social relationships are associated with an increased risk of developing CHD and stroke. Future studies are needed to investigate whether interventions targeting loneliness and social isolation can help to prevent two of the leading causes of death and disability in high-income countries.

**Study registration number:**

CRD42014010225.

## Introduction

Adults who have few social contacts (ie, who are socially isolated) or feel unhappy about their social relationships (ie, who are lonely) are at increased risk of premature mortality.^1^ The influence of social relationships on mortality is comparable with well-established risk factors, including physical activity and obesity.^2^ Yet, compared with our understanding of these risk factors, we know much less about the implications of loneliness and social isolation for disease aetiology.

Researchers have identified three main pathways through which social relationships may affect health: behavioural, psychological and physiological mechanisms.^3^ ^4^ Health-risk behaviours associated with loneliness and social isolation include physical inactivity and smoking.^5^ Loneliness is linked to lower self-esteem and limited use of active coping methods,^6^ while social isolation predicts decline in self-efficacy.^7^ Feeling lonely or being socially isolated is associated with defective immune functioning and higher blood pressure.^8^ ^9^ This evidence suggests that loneliness and social isolation may be important risk factors for developing disease, and that addressing them would benefit public health and well-being.

The aim of this study was to investigate the size of the association between deficiencies in social relationships and incident coronary heart disease (CHD) or stroke, the two greatest causes of burden of disease in high-income countries.^10^ We conducted a systematic review to answer the following primary question: are deficiencies in social relationships associated with developing CHD and stroke in high-income countries? Our secondary objectives included investigating whether loneliness or social isolation was differentially associated with incident heart disease and stroke, and whether the association between social relationships and disease incidence varied according to age, gender, marital status, socioeconomic position, ethnicity and health.

## Methods

This study followed the Centre for Reviews and Dissemination's Guidance for undertaking reviews in healthcare.^11^ A protocol was registered with the International Prospective Register of Systematic Reviews (registration number: CRD42014010225).^12^

### Study eligibility criteria

To meet inclusion criteria, studies had to investigate new CHD and/or stroke diagnosis at the individual level as a function of loneliness and/or social isolation. We defined CHD as encompassing the diagnoses listed under codes l20–l25 of the 10th revision of the International Statistical Classification of Diseases and Related Health Problems (ICD-10), and stroke as ICD-10 codes I60–69. We excluded studies where CHD or stroke diagnosis was not the first instance of diagnosis among participants, except where analyses controlled for previous events. We applied no other exclusion criteria regarding study population. Measures of social relationships met inclusion criteria for loneliness if they were consistent with its definition as a subjective negative feeling associated with someone's perception that their relationships with others are deficient.^13^ Measures of social isolation had to be consistent with its definition as a more objective measure of the absence of relationships, ties or contact with others.^14^ We focused on longitudinal studies in order to investigate the temporal relationships between loneliness or isolation and subsequent disease. Our purpose was to clarify the public health challenge posed by deficiencies in social relationships in high-income countries,^15^ so we excluded all other settings. We applied no language, publication type or date restrictions to inclusion.

### Search strategy and selection criteria

We searched 16 electronic databases for published and grey literature published up until May 2015: MEDLINE, EMBASE, CINAHL Plus, PsycINFO, ASSIA, Web of Science, Cochrane Library, Social Policy and Practice, National Database of Ageing Research, Open Grey, HMIC, ETHOS, NDLTD, NHS Evidence, SCIE and National Institute for Health and Care Excellence (NICE). Thesaurus and free text terms (eg, loneliness, social isolation, social relationships, social support, social network) were combined with filters for observational study designs and tailored to each database. The search strategy included no health terms, as it aimed to capture all disease outcomes, rather than focus on CHD and stroke. For the full electronic strategy used to search MEDLINE, see online supplementary appendix 1.

10.1136/heartjnl-2015-308790.supp1 Supplementary appendix 1

To complement the electronic search, we screened reference lists, searched for citations in Scopus (the largest database of abstracts and citations) and contacted topic experts identified through the UK Campaign to End Loneliness’ Research Hub.

After removing duplicates, two researchers independently screened titles and abstracts before assessing full records using a standardised screening sheet. Additional information was sought from authors when necessary (3 (60%) responded). When authors did not reply, we searched for information from related publications to inform our decision.

### Data extraction and quality assessment

Data were extracted into a standardised form by one researcher, and checked by a second. Study authors were contacted to obtain missing data.

Based on the Agency for Healthcare Research and Quality framework and taxonomy of threats to validity and precision,^16^ we selected the following domains as relevant for assessing studies: sampling bias, non-response bias, missing data, differential loss to follow-up, information error with regard to exposure and outcome measure, detection bias, confounding and study size. We identified age, gender and socioeconomic status as potential confounders (ie, factors correlated with exposure, predictive of outcome and not on the causal pathway).^17^ ^18^ No studies were excluded due to quality; instead, subgroup and sensitivity analyses were performed, to test the stability of findings according to internal validity.

### Quantitative synthesis

We hypothesised that social relationships were associated with disease incidence, and that this association may differ according to the dimension of relationships measured, and individual-level and contextual-level factors. A preliminary synthesis was developed by grouping study characteristics and results according to their measure of relationships. The majority of papers reported relative hazards of new diagnosis, comparing people with higher versus lower levels of loneliness or social isolation. Since incidence of disease was low (<10%) in the three studies reporting ORs, these estimates were approximated to relative risks.^19^ Where the lonely or isolated group was used as the reference, results were transformed to allow comparison across studies.

Patterns identified in the preliminary synthesis were formally investigated. Only papers for which an effect estimate and SE or CI were available (reported in the paper or provided by contacted authors), or could be calculated, contributed to this stage of the analysis. Where several papers reported results from the same cohort, we privileged the findings with the longest follow-up time. If a study included multiple measures of exposure and/or outcome, we selected the result relating to the most comprehensive measure. Where a study used statistical controls to calculate an effect size, we extracted data from the most complex model to minimise risk of confounding. All effect sizes were transformed to the natural log for analyses. Using Revman V.5.3 (Review Manager (RevMan) Version 5.3 [program]. Copenhagen: The Nordic Cochrane Centre, 2014), CHD and stroke effect estimates were plotted in separate forest plots, and heterogeneity between studies was assessed using the I^2^ statistic.

Potential sources of variation were explored with prespecified subgroup analyses. Since heterogeneity could not be explained and removed based on these analyses, but we deemed studies sufficiently similar to warrant aggregation, we combined results using random effects models. This approach allows for between-study variation, and is consistent with our assumption that the effects estimated in the different studies were not identical, since they investigated different dimensions of social relationships and derived from different populations.

Finally, sensitivity analyses were performed to test whether our overall results were affected by internal study validity and small-study effects. Contour-enhanced funnel plots for asymmetry were drawn using STATA V.12 (Stata Statistical Software: Release 12 [program]. College Station, TX: StataCorp LP, 2011). The limited number and the heterogeneity of studies did not support the use of tests for funnel plot asymmetry.^20^

## Results

**Figure 1:**
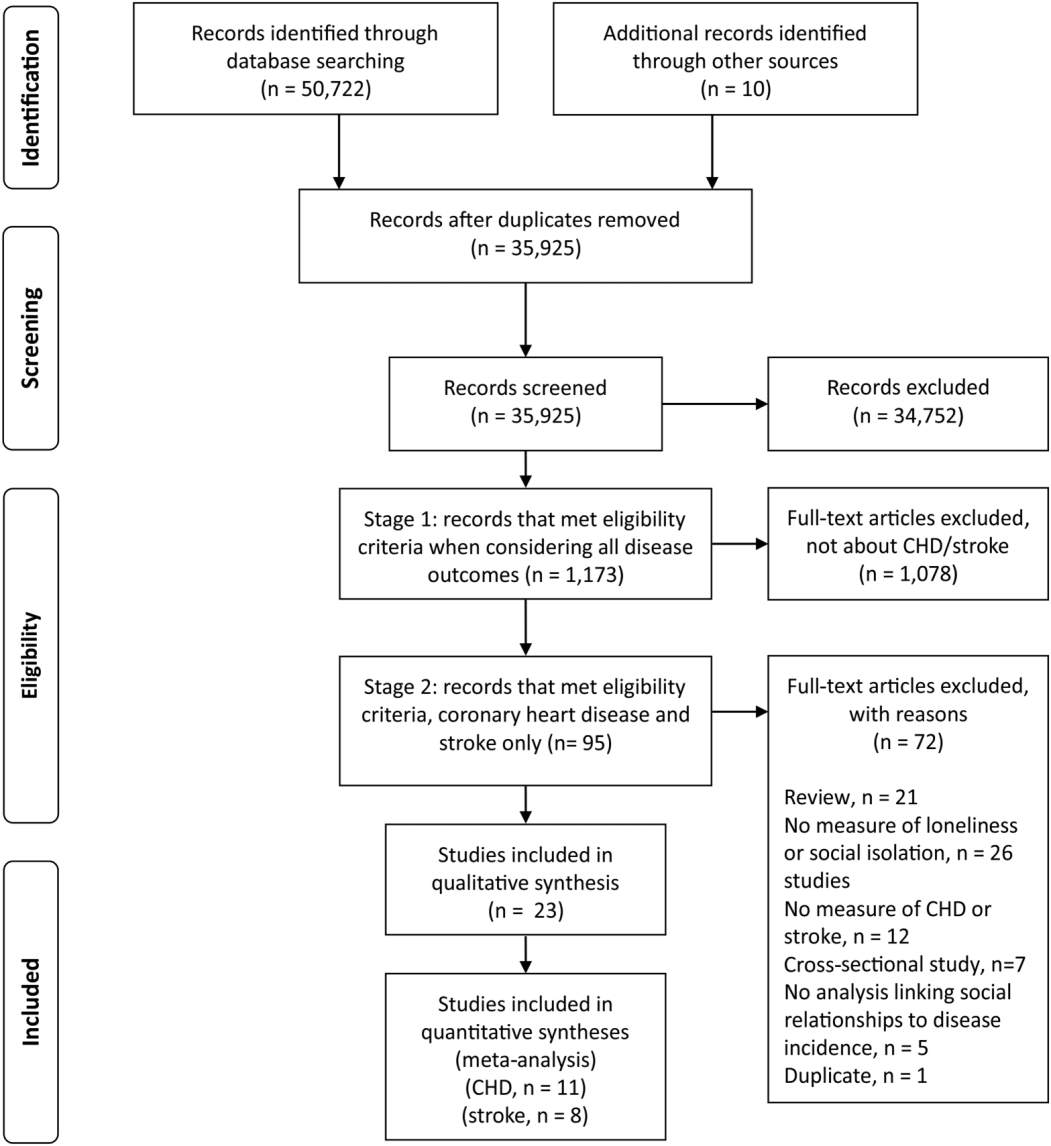
Preferred Reporting Items for Systematic Reviews and Meta-Analyses (PRISMA) flow diagram. CHD, coronary heart disease.

A total of 23 studies based on 16 cohorts were identified for inclusion in the review, after a two-stage process. See figure 1 for a flow diagram of the study selection process. Eleven studies on CHD and eight studies on stroke met inclusion criteria for the quantitative syntheses (ie, studies based on independent samples reporting data from which the natural log of the estimate and its SE could derived).

Table 1 summarises the descriptive characteristics of the evidence included in our review (see online supplementary appendix 2 for individual study characteristics).

10.1136/heartjnl-2015-308790.supp2 Supplementary appendix 2

**Table 1:** Characteristics of the included evidence

Population characteristics across included studies	
Total number of participants	181 006
Age of participants	18 and over
Breakdown of the population according to world region	Europe: 38% of participants North America: 33% of participants Asia (Japan and Asian Russia): 25% of participants Australia: 5% of participants
Study characteristics	
Baseline data collection years, range	1965–1996
Length of follow-up, range	3–21 years
Size, range	Between 98 and 47 713 subjects
Gender	All-male sample in nine papers^21–29^ All-female sample in six papers^30–35^ Mixed sample in eight papers^36–43^

### Assessment of loneliness and social isolation

Prevalence of loneliness or social isolation ranged from 2.8%^40^ to 77.2%.^31^ Three papers measured loneliness,^21^ ^30^ ^42^ 18 measured social isolation^22–43^ and two papers used a measure combining both dimensions.^34^ ^35^ The three papers on loneliness used different tools: a direct question asking about loneliness feelings during the day,^30^ a question on feelings of loneliness in the past week^42^ and a 13-item tool encompassing the perceived availability, adequacy or accessibility of social relationships.^21^ Across the 18 studies on social isolation, 11 tools were used: six studies used the Berkman–Syme Social Network Index,^44^ two studies used the 10-item Lubben Social Network Scale^45^ and the remainder used nine different tools on the availability and/or frequency of contacts. One cohort study used a measure combining social isolation and loneliness, the 11-item Duke Social Support Index, which asks about frequency of interaction and satisfaction with relationships.^46^

Loneliness and social isolation were predominantly treated as a categorical variable; two studies analysed them as continuous variables.^29^ ^42^ Only one study reported results based on measuring social relationships more than once.^42^

### Ascertainment of CHD and stroke

A total of 4628 CHD and 3002 stroke events were recorded across the 23 papers. Eighteen studies measured incident CHD and 10 measured stroke (five studies reported on both outcomes). Diagnosis was ascertained from medical records, death certificates or national registers in all but four studies. Others used self-report,^34^ ^35^ or telephone interviews with a nurse or physician.^33^ Two studies verified self-reported events against medical records.^29^ ^36^ ^38^ The majority of studies with a measure of CHD focused on myocardial infarction and/or CHD death (11/18). Four studies included angina pectoris within their measure of CHD and two presented results for angina separately. The remit of the CHD measure was unclear in one study.^43^

### Study validity

**Figure 2:**
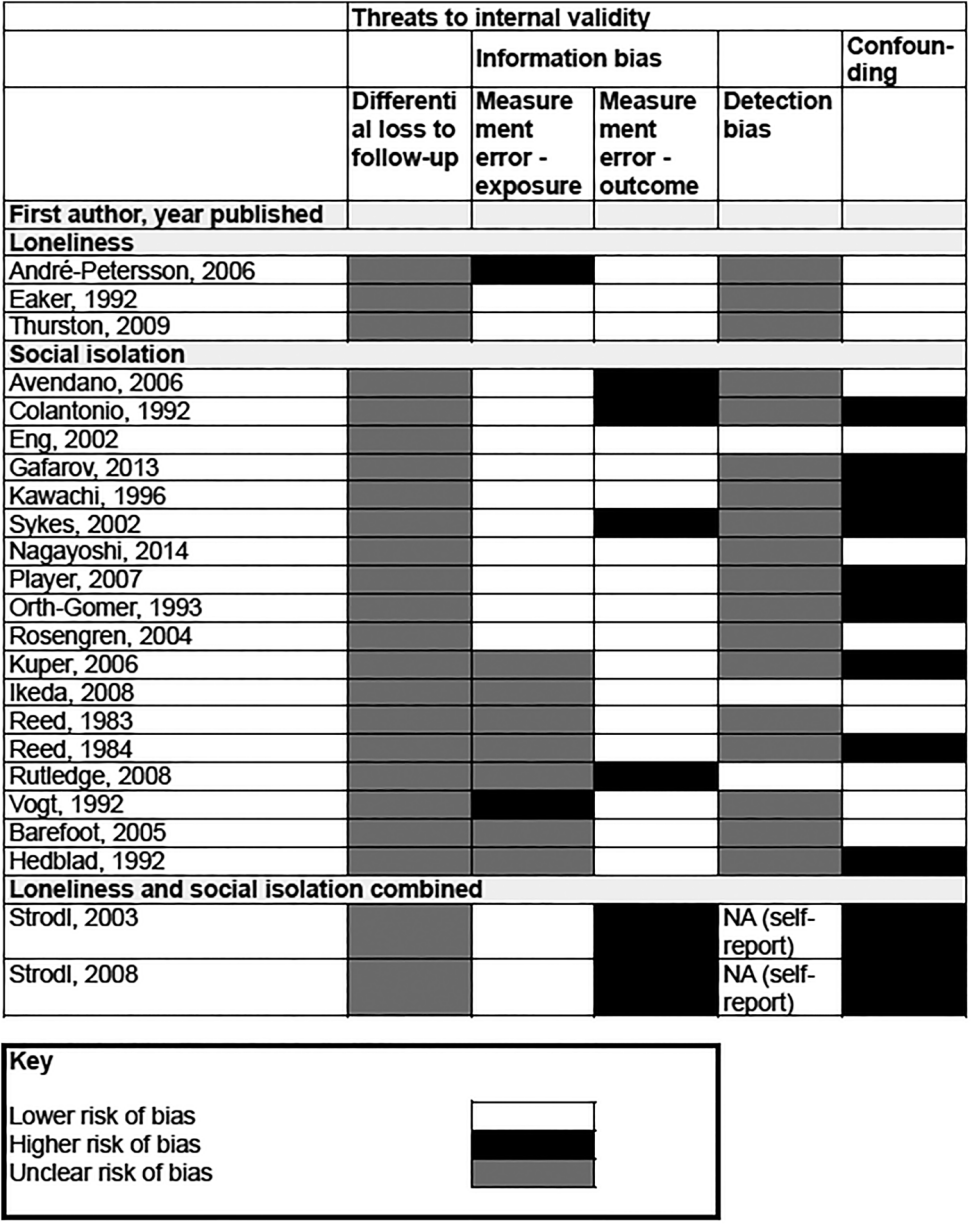
Internal validity. NA, not applicable.

Figure 2 summarises risk of bias across the studies included in our review (see online supplementary appendix 3 for details of criteria). For many of the instruments assessing social relationships, information on reliability and validity was limited (online supplementary appendix 4 displays detailed information on the validity and reliability of tools). Four cohorts (six articles) relied on subjects reporting new diagnosis for all or part of the outcomes measured, and were judged to be at greater risk of misclassification (see online supplementary appendix 2 for details of outcome assessment). Limited information on attrition and blinding of outcome assessment meant that susceptibility to differential loss to follow-up and detection bias was unclear. We note that the multiplicity of risk factors investigated and the differential length of follow-up suggest that outcome assessment is unlikely to have been influenced by knowledge of baseline information on social relationships.

10.1136/heartjnl-2015-308790.supp3 Supplementary appendix 3

10.1136/heartjnl-2015-308790.supp4 Supplementary appendix 4

The results reported in 12 papers were at lower risk of confounding, that is, analyses controlled or accounted for age, gender and socioeconomic status.^21^ ^22^ ^27^ ^28^ ^30^ ^33^ ^36^ ^37^ ^39^ ^40^ ^42^ ^43^ Four studies presented results from univariate analyses,^31^ ^34^ ^35^ ^41^ with a further study adjusting for age only.^26^ The remaining eight reports did not control for socioeconomic status, although in the case of the Health Professionals Follow-up Study the relative socioeconomic homogeneity of the sample may limit the impact of this omission.^22^ ^24^

### Loneliness, social isolation and CHD

**Figure 3:**
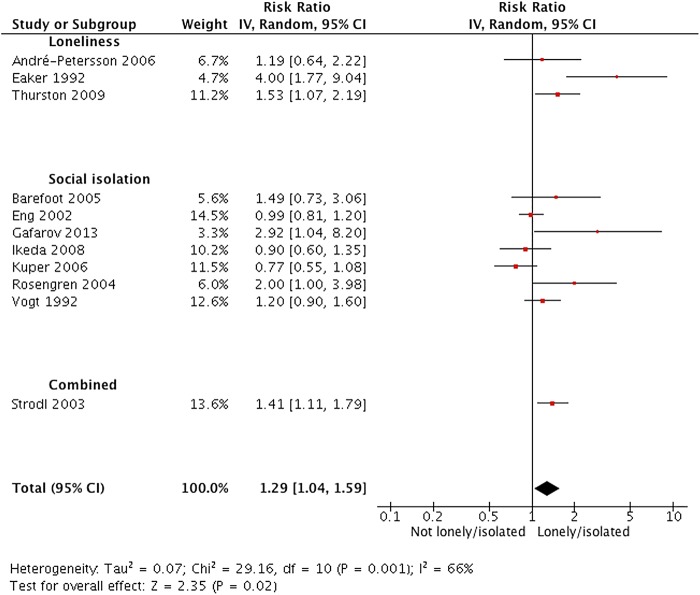
Forest plot of studies investigating incident CHD. CHD, coronary heart disease.

Across 11 studies (3794 events; one study did not report numbers) based on independent samples, the average relative risk of new CHD when comparing high versus low loneliness or social isolation was 1.29 (95% CI 1.04 to 1.59; see figure 3). We found evidence of heterogeneity within this comparison (I^2^=66%, χ^2^=29.16, df=10, p=0.001) and explored whether this could be explained by social relationship domain (loneliness vs social isolation), gender, risk of confounding and higher risk of bias due to exposure measurement error. We found no evidence that effects differed according to each subgroup (see online supplementary appendix 5). We were not able to explore other potential sources of heterogeneity due to limited information and study numbers.

10.1136/heartjnl-2015-308790.supp5 Supplementary appendix 5

### Social isolation and stroke

**Figure 4:**
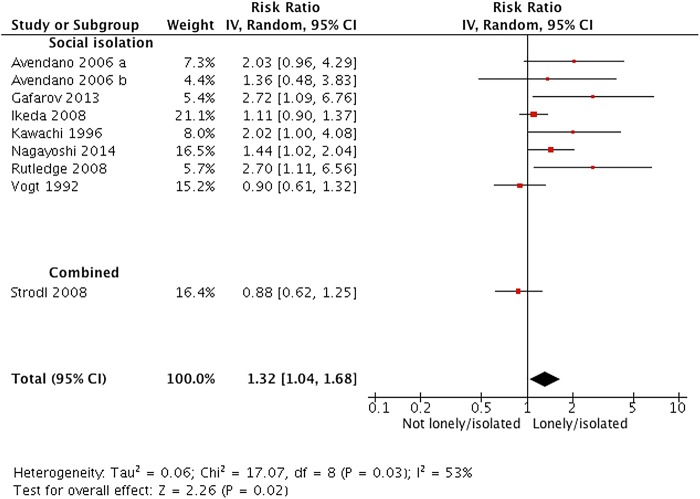
Forest plot of studies investigating incident stroke.

Across nine independent study samples (2577 events; one study did not report numbers), the average relative risk of stroke incidence was 1.32 (95% CI 1.04 to 1.68; see figure 4). Following confirmation of heterogeneity (I^2^=53%, χ^2^=17.07, df=8, P=0.03) we performed subgroup analyses according to risk of confounding and risk of bias due to outcome measurement error (there were too few studies to perform any other analyses). There was no evidence of effects differing according to subgroup (see online supplementary appendix 6); we had insufficient information to explore other potential sources of heterogeneity.

10.1136/heartjnl-2015-308790.supp6 Supplementary appendix 6

### Risk of bias across studies

To test whether our findings were sensitive to internal study validity, we compared results with and without studies at greater risk of bias. We found no evidence of a difference in the ratio of the relative risks for CHD and stroke according to study validity (see table 2).

**Table 2:** Sensitivity analyses

	Pooled estimate of the relative risk, based on all studies (95% CI) (number of effect estimates)	Without studies at greater risk of information bias (exposure)	Without studies at greater risk of information bias (outcome)	Without studies at greater risk of confounding	Without studies at greater risk of bias in at least one domain
Coronary heart disease	1.29 (1.04 to 1.59) (n=11)	1.34 (1.03 to 1.74) (n=9)	1.28 (1.01 to 1.63) (n=10)	1.34 (1.03 to 1.76) (n=7)	1.42 (1.00 to 2.01) (n=7)
Stroke	1.32 (1.04 to 1.68) (n=8)	1.42 (1.09 to 1.85) (n=7)	1.30 (0.98 to 1.71) (n=4)	1.34 (1.05 to 1.73) (n=6)	1.30 (0.98 to 1.71) (n=4)

**Figure 5:**
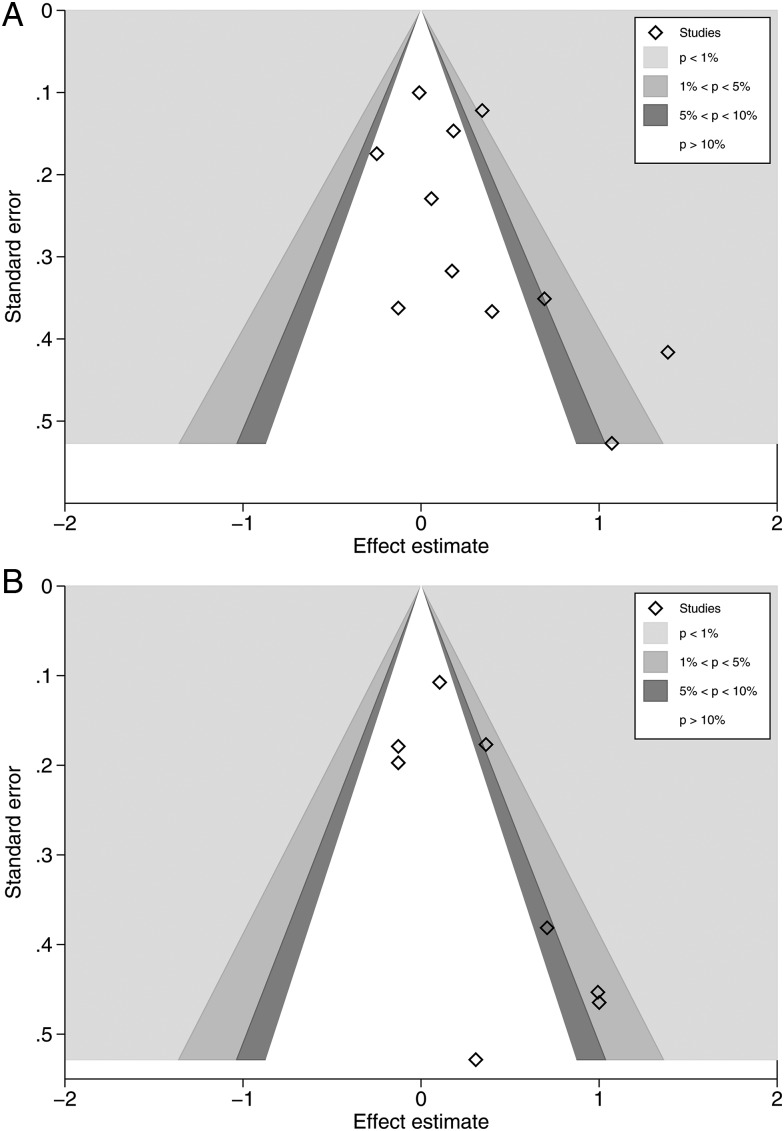
(A) Contour-enhanced funnel plot, coronary heart disease studies. (B) Contour-enhanced funnel plot, stroke studies.

Visual assessment of contour-enhanced funnel plots suggested that studies might be missing in areas of statistical significance (see figure 5A, B). Comparing fixed-effects and random-effects estimates, we found the random-effects estimate to be more beneficial (CHD: relative risk (RR) random-effects: 1.29, 95% CI 1.04 to 1.59, compared with RR fixed-effects: 1.18, 95% CI 1.06 to 1.31; stroke: RR, random-effects: 1.32, 95% CI 1.04 to 1.68, compared with RR fixed-effects: 1.19, 95% CI 1.03 to 1.36). This suggests the presence of small-study effects, which could be due to reporting bias. Although we found no evidence that study quality and true heterogeneity explained small-study effects in our review, these, along with chance, remain possible explanations.

### Additional studies

Seven papers with a measure of social isolation were excluded from quantitative synthesis since they either did not report data in a format suitable for pooling and/or shared data with other studies.^23^ ^25–27^ ^29^ ^38^ ^41^ Of the four papers that did not duplicate data from other studies, two reported results based on the Honolulu Heart Program: social isolation appeared to predict CHD but not stroke, in analyses adjusted for age, though the association disappeared in multivariate analysis.^26^ ^27^ In a univariate analysis of data from the Atherosclerosis Risk in Communities Study (USA) the Lubben Social Network score was not significantly associated with incident CHD among people with prehypertension.^41^ A further study found no evidence of an association between social isolation and CHD among men in France and Northern Ireland,^29^ although we note that this study controlled for depression, one of the possible pathways through which social isolation might lead to disease.

## Discussion

### Summary of findings and comparison with other work

Our review found that poor social relationships were associated with a 29% increase in risk of incident CHD and a 32% increase in risk of stroke. This is the first systematic review to focus on the prospective association between loneliness or social isolation and first occurrence of CHD or stroke.

Earlier reviews reported that cardiovascular disease (CVD) prognosis is worse among people with poorer social relationships.^1^ ^2^ Narrative reviews on social support and CHD have described an association with prognosis as well as incidence, but the strength of evidence was low.^47^ ^48^ A recent review of seven papers linked loneliness and social isolation to occurrence of CHD,^49^ but the effect on prognosis and incidence could not be disentangled.

We found an association between poor social relationships and incident CVD comparable in size to other recognised psychosocial risk factors, such as anxiety^50^ and job strain.^51^ Our findings indicate that efforts to reduce the risk of CHD and stroke could benefit from taking both loneliness and social isolation into account, as we found no evidence to suggest that one was more strongly related to disease incidence than the other. This is in line with other research linking subjective and objective isolation to hypertension, a risk factor for both stroke and CHD.^8^ ^9^

### Strengths and limitations

Our focus on longitudinal studies allowed us to comment on the direction of the relationship between social relationships and health, and avoid the problem of reverse causation. Pooling results from studies of CHD that measured loneliness and isolation allowed us to answer the broader question of whether deficiencies in social relationships are associated with disease incidence. We anticipated and explored heterogeneity where possible but found no statistical evidence that components of internal validity were associated with effect estimates.

Subgroup analyses specified a priori showed no difference between the association of loneliness or social isolation with CHD incidence, and we found no evidence across studies of differences between men and women. We found insufficient data to explore the relative effects of the quantity and quality of relationships, or study effect modifiers in depth. Seven of the estimates included in our meta-analyses (five CHD, two stroke) were extracted from studies where participants were of higher socioeconomic status and in better health than the target population. The role of deficiencies in social relationships may be greater among individuals under stress,^52^ and our results may underestimate the health-damaging implications of loneliness and social isolation among disadvantaged groups. Our review included some data collected from 1965; more recent strategies for CHD prevention may have modified the influence of loneliness and social isolation on disease incidence.

In common with other reviews of observational studies, we cannot infer causality from our findings, nor can we exclude confounding by unmeasured common causes, or reverse causation if deficiencies in social relationships are the result of subclinical disease. Publication bias is a concern in every review, and may lead us to overestimate the ‘true’ effect of poor social relationships. Conversely, our pooled effects could be a conservative estimate: most of the studies in this review statistically adjusted for factors that are likely to be on the causal pathway, such as depression or health-related behaviour.

### Implications

The main finding of our review, that isolated individuals are at increased risk of developing CHD and stroke, supports public health concerns over the implications of social relationships for health and well-being. Our work suggests that addressing loneliness and social isolation may have an important role in the prevention of two of the leading causes of morbidity in high-income countries.

A variety of interventions directed at loneliness and social isolation have been developed, ranging from group initiatives such as educational programmes and social activities, to one-to-one approaches including befriending and cognitive-behavioural therapy. These have primarily focused on secondary prevention, targeting people identified as isolated or lonely, but their effectiveness is unclear. Evaluative research is needed to investigate their impact on a range of health outcomes. Addressing health-damaging behaviours is also likely to be important, with lonely and isolated people more likely to smoke and be physically inactive, for example^5^ primary prevention strategies, such as promoting social networks or developing resilience, have received limited attention to date. Risk factors for loneliness and social isolation such as gender, socioeconomic position, bereavement and health status are well established^14^ ^18^ and hold the key to identifying people who may benefit from intervention.

Our findings suggest that tackling loneliness and isolation may be a valuable addition to CHD and stroke prevention strategies. Health practitioners have an important role to play in acknowledging the importance of social relations to their patients.^53 54^

**Key messages**

**What is already known on this subject?**

- People with poorer social relationships are at increased risk of premature death. The implications of social relationships for disease onset are unclear.

**What might this study add?**

- This systematic review of prospective longitudinal studies found that deficiencies in social relationships are associated with an increased risk of developing coronary heart disease and stroke of around 30%. This association is comparable in size to other recognised psychosocial risk factors, such as anxiety and job strain.

**How might this impact on clinical practice?**

- Efforts to reduce cardiovascular disease incidence need to consider loneliness and social isolation.
