# Supplementary material for: Loneliness and social isolation as risk factors for coronary heart disease and stroke: systematic review and meta-analysis of longitudinal observational studies
Source: Heart. 2016 Apr 18;102(13):1009–16. doi: 10.1136/heartjnl-2015-308790 (PMC4941172; doi:10.1136/heartjnl-2015-308790)
Supplement: Supplementary appendix 2 [file heartjnl-2015-308790supp_appendix2.pdf]

Appendix 2 Overview of studies included in our review

Studies are grouped according to the dimension of social relationships they investigated (loneliness, social isolation or a combination of both); the measure of social relationships used (e.g. sudies using the Berkman-Syme Social Network Index are grouped together); and the datasets used (i.e. studies reporting data from the same dataset, e.g. the Established Populations for the Epidemiologic Studies of the Elderly Study, are grouped together).

**First author & year published**	**Data source & country**	**Data collection dates**	**Population group (number of participants)**	**Age of study subjects at baseline**	**Follow-up period - mean, median and/or range**	**Outcome measure**	**Number of events**	**Covariates adjusted for**	**Main results for our review**
**Studies with a measure of loneliness**									
André-Petersson, 2006	Men Born in 1914 Study, Sweden	1982-1983 to 1996	Men born in 1914 and living in Malmö in 1982/3, who showed adaptive behaviour in a stressful situation (n=208)	67-68	Mean: 10.3 years	Myocardial infarction (MI), fatal or non-fatal; register based	43	Prevalent CHD (previous experience of MI or angina pectoris), use of antihypertensive drugs, socio-economic status (SES), s-triglycerides	Cox proportional hazards model. Comparing subjects with unsatisfactory (lonely) v. satisfactory social support (not lonely), Hazard ratio (HR): 1.19, 95% CI: 0.64-2.22
Eaker, 1992	Framingham Heart Study, USA	1965-1967 to 1987	Female homemakers living in Framingham, Massachusetts in 1948 and completed a 300-item psychosocial questionnaire (n=353)	45 to 64	Study length: 20 years	MI or coronary death, both sudden and not sudden; medical records.	30	Age, systolic blood pressure, ratio of serum total cholesterol to high-density lipoprotein cholesterol, diabetes, cigarettes, body mass index (BMI)	Cox proportional hazards model. Comparing lonely v. not lonely subjects, HR: 4.0, 95% CI: 1.8-9.2

Thurston, 2009	National Health and Nutrition Survey, USA	1971-1975 to 1992	Civilian non-institutionalized men (44%) and women (56%) who were administered the Center for Epidemiological Studies-Depression Scale (CESD) at baseline (n=2,616).	Mean: 44.8 years. Range: 25-74.	Mean (SD): 14.9 years (5.1); range: 0–18.9 years	CHD, ICD-9 codes 410 to 414; home discharge reports and death certificates.	357	Age, race, education, income, marital status, hypertension, diabetes, cholesterol, physical activity, smoking, alcohol use, systolic and diastolic blood pressures, body mass index, depressive symptoms	Cox proportional hazards model. Whole sample: comparing subjects with high v. low levels of loneliness, HR: 1.53, 95% CI 1.07–2.21. Men: comparing subjects with high v. low levels of loneliness, HR: 0.88, 95% CI: 0.43–1.78. Women: comparing subjects with high v. low levels of loneliness, HR = 1.81, 95% CI: 1.20–2.94.
**Studies with a measure of social isolation**									
Avendano, 2006	Established Populations for the Epidemiologic Studies of the Elderly Study (EPESE, New Haven sample), USA	1982 to 1994	Men and women living in New Haven, Connecticut, in 1982, in one of 3 housing strata: 1) public elderly housing that is age and income restricted, 2) private elderly housing that is age restricted, and 3) private community housing and apartments. (n=2,250)	65+	Study length: 12 years	Stroke, fatal or non-fatal; self-report of diagnosis, obituaries, surveillance of hospital admissions and annual interviews with next of kin. Records were matched to the National Death Index and death certificates.	270	Age, sex, race, education, income	Cox proportional hazards model. Aged 65-74: comparing people who scored 1 or less v. scoring higher on the social network index, HR: 2.03, 95% CI: 0.96–4.28. Aged 75+: comparing people who scored 1 or less v. scoring higher on the social network index, HR: 1.36, 95% CI: 0.48–3.81.

Colantonio, 1992	Established Populations for the Epidemiologic Studies of the Elderly Study (EPESE, New Haven sample), USA	1982 to 1988	Men (41%) and women (59%) aged 65 and over, living in New Haven, Connecticut, in 1982, in one of 3 housing strata: 1) public elderly housing that is age and income restricted, 2) private elderly housing that is age restricted, and 3) private community housing and apartments. (n=2,604)	Mean: 74	Study length: 7 years	Stroke; hospital admission records, death certificates, Health Care Financing Administration data, self-reported strokes from annual contacts - verified against hospital records, using ICD-9 codes 431, 432.9, 433.0-434.9 and 436-437.1.	167	Age, sex, housing stratum, hypertension, diabetes, physical function, smoking.	Cox proportional hazards model. When the social network variable was added to the multivariate model, X^2= 0.15, df=1, p-value=0.6995.

Eng, 2002	Health Professionals Follow-up Study, USA	1988 to 1998	Male health professionals aged 40 to 75 in 1986 (n=28,369)	Mean: 55.2 years; range: 42-77	Study length: 10 years	4 outcomes: nonfatal MI, fatal MI, sudden cardiac death, combined measure of total CHD(i.e. all incident first events of nonfatal myocardial infarction, fatal coronary heart disease, sudden cardiac death, coronary artery bypass graft surgery, and angioplasty); medical records, death certificates, interviews with next of kin.	1,816 incident cases of total coronary heart disease, including: 618 cases of nonfatal myocardial infarction, 142 cases of fatal coronary heart disease (excluding sudden cardiac death), and 97 sudden cardiac deaths.	Age (5-year categories), time period (1988–1990, 1990–1992, 1992–1994, 1994–1996, 1996–1998), occupation in 1986, smoking history (never, past, and current in categories of 1–14, 15–24, and ≥25 cigarettes/day), daily alcohol intake (0, 0.01–9.9, 10–19.9, 20–29.9, and ≥30 g/day), quintiles of body mass index, quintiles of physical activity, routine physical examination in the last 2 years (yes/no), ability to climb several flights of stairs (yes/no), ability to do heavy housework (yes/no), employment status (full time, part time, retired, disabled), history of hypertension, diabetes, high serum cholesterol, history of myocardial infarction in a parent aged <60 years (yes/no) in 1986, quintiles of energy-adjusted intakes of total fat, saturated fat, folate, and fiber, multivitamin and vitamin E supplement use (yes/no).	Cox proportional hazards model. Nonfatal myocardial infarction: comparing subjects with a low v. high level of social network, HR: 1.11, 95% CI: 0.80, 1.53. Fatal coronary heart disease: comparing subjects with a low v. high level of social network, HR: 1.82, 95% CI: 1.02, 3.23. Sudden cardiac death: comparing subjects with a low v. high level of social network, HR: 0.71, 95% CI: 0.28, 1.81. Total coronary heart disease: comparing subjects with a low v. high level of social network, HR: 0.99, 95% CI: 0.81, 1.20.

Kawachi, 1996	Health Professionals Follow-up Study, USA	1988 to 1992	Male health professionals aged 40 to 75 in 1986 (n=32,624)	Range: 42-77	Study length: 4 years	8 outcomes: total stroke, fatal stroke, nonfatal stroke, total CHD, fatal CHD, nonfatal CHD, sudden cardiac death, non-sudden cardiac death; medical records supplemented by correspondence and telephone interviews.	104 incident cases of stroke (91 non-fatal, 13 fatal) and 403 cases of incident coronary heart disease (275 non-fatal myocardial infarction, 128 cases of fatal coronary heart disease)	Age, time-period, smoking status, history of hypertension, diabetes mellitus, hypercholesterolemia, diagnosis of angina pectoris, deciles of BMI, parental history of MI before age 60, daily alcohol intake, tertiles of physical activity	Cox proportional hazards model. Total stroke: comparing subjects with a low v. high level of social network, HR: 2.02, 95% CI: 1.00, 4.08. Fatal stroke: age-adjusted model only, comparing subjects with a low v. high level of social network, HR: 3.64, 95% CI: 0.78, 16.9. Nonfatal stroke: comparing subjects with a low v. high level of social network, HR: 1.86, 95% CI: 0.85, 4.06. Total CHD: comparing subjects with a low v. high level of social network, HR: 1.14, 95% CI: 0.74, 1.73. Fatal CHD: comparing subjects with a low v. high level of social network, HR: 1.42, 95% CI: 0.72, 2.81. Nonfatal CHD: comparing subjects with a low v. high level of social network, HR: 1.00, 95% CI: 0.58, 1.71. Sudden cardiac death: comparing subjects with a low v. high level of social network, HR: 0.68, 95% CI: 0.16, 2.96. Non-sudden cardiac death: comparing subjects with a low v. high level of social network, HR: 1.89, 95% CI: 0.87, 4.13.
Gafarov, 2013	Multinational Monitoring of Trends and Determinants in Cardiovascular Disease (MONICA) - Psychosocial Study, Russia	1994 to 2010	Women aged 25-64 in 1994 living in one of the Novosibirsk districts, Western Siberia (n=870)	Range: 25-64	Study length: 16 years	2 outcomes: MI, stroke; register, medical records and death certificates.	MI: 15; stroke: 35	None reported	Cox proportional hazards model. Across the whole sample, MI: comparing subjects with low v. higher levels of social network, HR: 2.92, 95% CI: 1.040-8.208. Stroke: comparing subjects with low v. higher levsl of social network, HR: 2.72, 95% CI:1.094-6.763; p<0.05. In the 55-64 age group, MI: comparing subjects with low v. higher levels of social network, HR: 5.9, 95% CI: 1.534-22.947.<br>Focus on close contacts: MI: comparing subjects with low v. higher levels of close social network, HR :4.9, 95% CI: 1.108-21.762. Stroke: comparing subjects with low v. higher levels of social network, HR: 4.1, 95% CI: 1.193-14.055.

Sykes, 2002	Prospective Epidemiological Study of Myocardial Infarction (PRIME) Study, Northern Ireland and France	1991 to 1994	Men aged 50 to 59 in 1991-1994 and initially free from any cardiovascular disease (n=9,758)	Range: 50-59	Study length: 5 years	2 outcomes: 'hard CHD'(i.e. subjects who had died from CHD or had at least one non-fatal MI), total AP (i.e. angina pectoris or unstable angina); postal questionnaire followed-up by hospital or general practitioner notes, and death certificates.	Hard CHD: 163; total AP: 154.	Hostility, competitiveness, depression, impatience	Logistic regression. Hard CHD, full model: X2 = 7.389, df = 5, p-value: not significant. Total AP, full model: X2 = 16.242, df = 5, p = 0.006. The authors report that only depression and hostility contributed to the effect.
Nagayoshi, 2014	Atherosclerosis Risk in Communities Study, USA	1990-1992 to 2010	Men (44%) and women (56%) aged 45-64 in 1987-1989, living in one of 4 communities: Washington County, MD; suburban Minneapolis, MN; Forsyth County, NC; and Jackson, MS (n=13,686)	Mean: 57; range: 46-69	Median: 18.6 years; maximum: 20.9 years	2 outcomes: stroke, and ischemic stroke only; phone calls, surveillance of hospital discharges, death certificates - checked against medical records.	905 incident strokes, of which ischemic: 804	Age, sex and race, socioeconomic status (education attainment, income, occupation), marital status, behavioural risk factors (smoking status, alcohol drinking, physical activity), major stroke risk factors (hypertension, diabetes mellitus, low-density lipoprotein, high-density lipoprotein, lipid-lowering medication use, body mass index)	Cox proportional hazards model. Stroke: conparing subjects with small v. larger network, HR: 1.44, 95% CI: 1.02–2.04. Ischemic stroke only: comparing subjects with small v. large social networks, HR: 1.41, 95% CI: 0.98-2.03.

Player, 2007	Atherosclerosis Risk in Communities Study, USA	1990-2 to 1996-1998	Men (48.3%) and women (51.7%) aged 45-64 in 1987-1989, living in one of 4 US communities and with blood pressures in the prehypertension range (120 to 139 mm Hg systolic or 80 to 89 mm Hg diastolic) (n=2,334)	Range: 48-67; 50.4% sample were aged 48-57, 49.5% aged 58-67 years	Range: 4-8 years	Progression from prehypertension to CHD or CHD death; hospital discharge summaries, death certificates, supplmented with information from interviews with next of kin and corroners' or medical examiners' reports for out-of-hospital causes and dates of death.	262	None	χ2 analysis. P-value associated with χ2 in bivariate analysis re. social networks = 0.220
Rosengren, 2004	Men born in 1933 Study, Sweden	1983 to 1998	Men born in 1933 in Gothenborg (n=741)	49-50	Study length: 15 years	CHD (MI; coronary revascularization; acute hospitalization with discharge diagnosis of angina); hospital registers.	92	Smoking, physical activity, serum cholesterol, serum triglyceride, systolic blood pressure, BMI, diabetes, family history of CHD	Cox proportional hazards modelling. Comparing subjects with high v. low levels of integration, HR = 0.45, 95% CI: 0.24-0.84
Orth-Gomer, 1993	Men born in 1933 Study, Sweden	1983 to 1988-9	Men born in 1933 in Gothenborg (n=736)	49-50	Study length: 6 years	CHD, including fatal and non fatal MI and sudden and non sudden coronary death; hospital records, death certificates, national cause-specific death register.	25	Serum cholesterol, BMI, smoking, treatment for hypertension, diabetes, physical activity	Multiple logistic regression. Comparing subjects in the lower v. upper quartiles of social integration, OR = 3.8, 95% CI: 1.1-13.9; regression coefficient = -0.656, SE = 0.327, p=0.04.

Kuper, 2006	Women's Lifestyle and Health Cohort Study, Sweden	1991-1992 to 2002	Women aged 30–50 residing in the Uppsala health care region (n=47,713)	Mean (SD): 40.3 years (5.8); range: 30-50	Mean: 135 months, i.e. 11 years and 3 months	Fatal and non-fatal MI; national hospital discharge and death registers.	210	Age, cigarette smoking, exercise, alcohol consumption, weight, height, diabetes, high blood pressure	Cox proportional hazards modelling. Comparing subjects with the highest v. lowest social support, HR = 1.3, 95% CI: 0.9-1.8
Ikeda, 2008	Second cohort of the Public Health Center-Based Prospective Study, Japan	1993-1994 to 2004	Men (47.5%) and women (52.5%) aged 40 to 69 years in 1993-1994 and living in one of 13 administrative districts (n=44,152)	Mean: 53.6; range: 40-69	Mean: 10.7 years	4 outcomes: nonfatal MI, fatal MI, nonfatal stroke, fatal stroke; medical records, letter and telephone follow-up, death certificates.	Nonfatal MI: 301; fatal MI: 191, nonfatal stroke: 1,057; fatal stroke: 327	Age (years), smoking status (never, former, or current), ethanol intake (nondrinkers and former drinkers, less than weekly, <150 g/wk, or ≥150 g/wk), body mass index (kg/m2 in quartiles), leisure time sports activity (<1 day/mo, 1 to 3 days/mo, or ≥1cday/wk), perceived stress (less, moderate, or high), occupation	Cox proportional hazards model. Nonfatal MI: comparing subjects with low v. very high levels of support, HR = 0.90, 95% CI: 0.60-1.35; comparing men with low v. very high levels of support, HR = 1.06, 95% CI: 0.68-1.67; comparing women with low v. very high levels of support, HR = 0.55, 95% CI: 0.19-1.57. Fatal MI: comparing subjects with low v. very high levels of support, HR = 1.00, 95% CI: 0.61-1.63; comparing men with low v. very high levels of support, HR = 1.12, 95% CI: 0.65-1.94; comparing women with low v. very high levels of support, HR = 0.58, 95% CI: 0.17-1.99. Nonfatal stroke: comparing subjects with low v. very high level of support, HR = 1.11, 95% CI: 0.89-1.37; comparing men with low v. very high level of support, HR = 1.09, 95% CI: 0.84-1.43; comparing women with low v. very high level of support, HR = 1.22, 95% CI: 0.85-1.74. Fatal stroke: comparing subjects with low v. very high level of support, HR = 1.45, 95% CI: 1.00-2.10; comparing men with low v. very high level of support, HR = 1.59, 95% CI: 1.01-2.51; comparing women with low v. very high level of support, HR = 1.25, 95% CI: 0.63-2.46.
Reed, 1984	Honolulu Heart Program Study, USA	1971 to 1979	Men of Japanese ancestry born between 1900 and 1919 and living in Oahu, Hawaii, in 1965 (n=4,251)	Range: 51-72	1971 to 1979	2 outcomes: CHD (MI and CHD deaths), stroke; hospital and medical records, postmortem data.	CHD: 155; stroke: 98	Age	Multiple logistic regression analysis. CHD: comparing subjects with low v. high networks, p-value ≤0.05. Stroke: comparing subjects with low v. high networks, p-value >0.05.

Reed, 1983	Honolulu Heart Program Study, USA	1971 to 1979	Men of Japanese ancestry born between 1900 and 1919 and living in Oahu, Hawaii, in 1965 (n=4,389)	Range: 51-72	1971 to 1979	4 outcomes: nonfatal MI, fatal MI, angina, and total CHD (MI+angina); hospital discharge and mortality records.	Total CHD: 218; nonfatal MI: 95; fatal MI: 76; angina: 47	Age, systolic blood pressure, serum cholesterol, serum glucose, serum uric acid, forced vital capacity, BMI, physical activity index, cigarettes/day, alcohol consumption, complex carbohydrate, socioeconomic status	Multiple logistic regression: coefficients associated with social network scores, CHD: beta = -0.0836, p-value > 0.5 nonfatal MI: beta = -0 0576, p-value > 0.5 fatal MI: beta = -0 0505, p-value > 0.5 angina: beta = -0.1348 , p-value > 0.5
Rutledge, 2008	Women's Ischemia Syndrome Evaluation (WISE) Study, USA	1996 to 2005	Women referred for a coronary angiogram to evaluate suspected myocardial ischemia (n=629)	Median (SD): 59.6 (11.6); aged 18+	Mean: 5.9 years	Stroke; inquiry by nurse or physician, death certificates.	32	Age, education history, ethnicity, Beck Depression Inventory scores, diabetes, smoking, dyslipidemia and hypertension histories, waist-circumference, coronary artery disease severity score	Cox proportional hazards modelling. Comparing subjects with low v. high social network index scores, HR= 2.7, 95% CI: 1.1-6.5.
Vogt, 1992	Health maintenance organization, USA	1970-1971 to 1986	Men (46.1%) and women (53.9%) enrolled for at least two years with a managed care health maintenance organization in Portland, Oregon (n=2,396)	15.5% aged 18-29; 30.25 aged 30-44; 36.8% aged 45-64; 17.5% aged 65+	Length of study: 15 years	2 outcomes: CHD, stroke; medical care and mortality records.	NR	Age, sex, socioeconomic status, current and former smoking, subjective health status	Cox proprotional hazards modelling. CHD: Comparing subjects in the low v. high tertiles of social network size, HR = 1.2, 95% CI: 0.9-1.6, p-value = 0.26. Stroke: Comparing subjects in the low v. high tertiles of social network size, HR = 0.9, 95% CI: 0.6-1.3, p-value = 0.58.
Barefoot, 2005	Copenhagen City Heart Study, Denmark	1991–1994 to 1997.	Men (44.3%) and women (55.7%) living in Copenhagen (n=9,460)	Mean: 57.5; range: 21-93	Mean: 5.7 years; maximum: 7.2 years	CHD; national health and hospital discharge registers.	427	Age, gender, education,BMI, family history of coronary disease, systolic blood pressure, glucose, high density lipoprotein cholesterol, total cholesterol, smoking, alcohol consumption, physical activity, self-rated health	Cox proportional hazards modelling. Comparing people with 6-7 contacts to 0-1 contacts, HR = 0.67, 95% CI: 0.33-1.39. Comparing people with 5 intimate contacts to no contact, HR = 0.39, 95% CI: 0.14-1.06.

Hedblad, 1992	Men Born in 1914 Study, Sweden	1982-1983 to 1987	Men born in 1914 and living in Malmö in 1982/3 who participated in a long-term ECG recording and had at least one episode of ST segment depression (n=98)	67-68	Mean: 53.1 months i.e. just over 4 years and 5 months	CHD; infarct and mortality registers.	17	Previous IHD, hypertension, hyperlipidaemia, obesity, high alcohol consumption, current smoking and low to moderate physical activity, social support and social influence indices, marital status	Multiple logistic regression. Comparing subjects with low v. high contact frequency, RR = 1.2, 95% CI: 0.3-4.9; regression coefficient = 0.193, SE = 0.713.
**Studies with a measure combining loneliness and social isolation**									
Strodl, 2003	Australian Longitudinal Study on Women's Health, Australia	1993 to 1996	Women aged 70-75 identified via national medicare database (n=6,994)	Range: 70-75	Study length: 3 years	CHD; self-report.	503	None	Logistic regression. Comparing subjects with low to fair v. very high social support, OR = 1.41, 95% CI 1.11-1.79, p-value ≤ .01.
Strodl, 2008	Australian Longitudinal Study on Women's Health, Australia	1993 to 1996	Women aged 70-75 identified via national medicare database (n=8,907)	Range: 70-75	Study length: 3 years	Stroke; self-report.	174	None	Logistic regression. Comparing subjects with low to fair v. very high social support, OR = 0.88, 95% CI: 0.62-1.25.
