# Supplementary material for: Loneliness and social isolation as risk factors for coronary heart disease and stroke: systematic review and meta-analysis of longitudinal observational studies
Source: Heart. 2016 Apr 18;102(13):1009–16. doi: 10.1136/heartjnl-2015-308790 (PMC4941172; doi:10.1136/heartjnl-2015-308790)
Supplement: Supplementary appendix 3 [file heartjnl-2015-308790supp_appendix3.pdf]

Appendix 3 Criteria for assessment of risk of bias

Domain	Lower risk of bias	Higher risk of bias	Unclear risk of bias
**Differential loss to follow-up**	Subjects lost to follow-up did not significantly differ from the rest of the sample.	There were significant differences in characteristics likely to increase risk of bias between the baseline data reported for the whole sample and the baseline data of subjects lost to follow-up.	No information available
**Measurement error - exposure**	Available data suggest that the tool used to measure loneliness and/or social isolation was comparatively valid and reliable.	Loneliness and/or social isolation were assessed using a tool that was of limited validity and/or reliability.	No information available
**Measurement error - outcome**	Measure based on information from medical records, registers and/or death certificates.	Reliance on self-report of diagnosis.	No information available
**Detection bias**	Assessors were blinded to exposure.	Assessors were not blinded to exposure.	No information available
**Confounding**	Studies controlled for CVD risk factors correlated with loneliness/social isolation, i.e. gender (in mixed samples), age, socio-economic status. Note that measures relating to health (e.g. diabetes, health-behaviours) are not included in this list because of them potentially being on the causal pathway.	Studies did not control for age, gender and socio-economic status.	No information available
