# Supplementary material for: Loneliness and social isolation as risk factors for coronary heart disease and stroke: systematic review and meta-analysis of longitudinal observational studies
Source: Heart. 2016 Apr 18;102(13):1009–16. doi: 10.1136/heartjnl-2015-308790 (PMC4941172; doi:10.1136/heartjnl-2015-308790)
Supplement: Supplementary appendix 4 [file heartjnl-2015-308790supp_appendix4.pdf]

Appendix 4 **Measurement and prevalence of loneliness and social isolation in the studies included in our review**

Studies are grouped according to the dimension of social relationships they investigated (loneliness, social isolation or a combination of both); the measure of social relationships used (e.g. sudies using the Berkman-Syme Social Network Index are grouped together); and the datasets used (i.e. studies reporting data from the same dataset, e.g. the Established Populations for the Epidemiologic Studies of the Elderly Study, are grouped together).

Record ID (first author & year)	Term used in the paper	Measurement	Nber of items	Scoring and categorisation	Prevalence of loneliness/isolation	Comments re. reliability, validity, responsiveness and/or interpretability	How many times were social relationships measured?	Effect estimates re. incident CHD and/or stroke
**Loneliness**								
André-Petersson, 2006	Social support	Combination of 3 sets of questions:<br>a) questions on the availability of emotional support: - Do you have any friends or relatives who you like very much and who like you very much?<br>- Do you have any really close friends with whom you feel intimate and with whom you can discuss anything?<br>- If you have continued to work, is it because you want to feel that you are a valuable and important person?<br>- When you have personal problems of any kind, do you have any close friend or relative to whom you can turn to discuss your problems? ,<br><br>b) questions on the adequacy of emotional support: - How often do you feel lonely?<br>- Do you have the feeling that people appreciate what you do?<br>- Do you have enough good friends to be with?<br>- Do you think that you see your children too often or too rarely?<br><br>c) questions about access to informal and material support:<br>- Is there anyone in your neighbourhood from whom you can borrow things or exchange services?<br>- Is there anyone in your	13	For each set of questions, answers were dichotomised based on a cutoff set at approximately the lowest 30th percentile. Subjects who scored low in any of the three domains (availability, adequacy or access) were considered to have an unsatisfactory level of social support; 2 categories: satisfactory v. unsatisfactory social support.	22.5% of subjects were classed as having unsatisfactory levels of social support, i.e. they scored low in one domain. 8.2% scored low in 2 domains, 3.4% scored low in 3 domains.	Hanson & Östergren (1987) reported that answers were generally skewed toward the positive side. Re. interpretability, note that subjects were classed as having social support if they scored low in one domain, regardless how they scored in the other domains. It was therefore possible for subjects reporting high access to support to be classed as having low social support because of scoring low in the adequacy domain, making the distinction between satisfactory and unsatisfactory levels of support difficult to interpret.	Once, at baseline	Comparing subjects with unsatisfactory (lonely) v. satisfactory social support (not lonely), Hazard Ratio (HR for incident CHD): 1.19, 95% CI: 0.64-2.22

		neighbourhood from whom you can get help if you fall ill?<br>- If you need help with something for 24hr, do you have anyone you can ask? (other than wife)<br>- Do you know anyone who can help you to write an official letter or to appeal against a decision made by some authority?<br>- Do you know where to go in order to get help to write an official letter or to appeal against a decision made by some authority?						
Eaker, 1992	Loneliness	'Are you lonely during the day?' Answer: yes or no	1	Subjects who answered yes to the loneliness question were classed as lonely; 2 categories: lonely v. not lonely.	Not reported	Direct question may deter participants from admitting to a socially stigmatised feeling, although there is evidence of similar questions correlating with the complex UCLA Loneliness Scale (Russell et al., 1978).	Once, at baseline	Comparing lonely v. not lonely subjects, HR for incident CHD: 4.0, 95% CI: 1.8-9.2
Thurston, 2009	Loneliness	Question taken from the Center for Epidemiologic Studies of Depression scale: participants were asked to rate the statement: "I feel lonely" on a 4-point scale: Rarely or none of the time (<1 day), some or a little of the time (1–2 days), occasionally or a moderate amount of the time (3–4 days), or most of the time (5-7 days) in the past week.	1	Scores were categorized as low (<1 day), medium (1–2 days), and high (3–7 days). The CESD was administered a second time, and answers were used with the baseline responses to characterize loneliness across the two time points. Subjects reporting low loneliness at both interviews were classed as low loneliness, those reporting high loneliness at either interview were classed as high loneliness, and the remainder were classed as having moderate levels of loneliness.Two analyses were performed: one where loneliness was split into three categories, and one where the loneliness score was treated as a	241 subjects (9.2%) were classed as having high levels of loneliness, 409 (15.6%) as medium and 1,966 (75.2%) as low	Direct question may deter participants from admitting to a socially stigmatised feeling, although there is evidence of similar questions correlating with the complex UCLA Loneliness Scale (Russell et al., 1978). The 'medium loneliness' category is difficult to interpret given that it includes subjects with moderate levels of loneliness at both time points as well as individuals who fluctuated from high to low loneliness, or vice versa.	Twice, second time on average 8.2 years after baseline (SD = 0.6; range = 6.7–9.7)	Comparing subjects with high v. low levels of loneliness, HR for incident CHD: 1.53, 95% CI 1.07–2.21.<br>Comparing men with high v. low levels of loneliness, HR for incident CHD: 0.88, 95% CI: 0.43–1.78.<br>: Comparing women with high v. low levels of loneliness, HR for incident CHD: 1.81, 95% CI: 1.20–2.94.

				continuous variable.				
**Social isolation**								
Avendano, 2006	Social network	Social Networks Index (SNI) developed by Berkman and Syme: 1) Have you ever been married? If so, are you now married, separated, divorced or widowed? 2) How many close friends do you have? How many relatives do you have that you feel close to? How many of these friends or relativesdo you see at least once a month? 3) 'Do you belong to any of these kinds of groups? Church/social or recreational group/labour union, commercial group or professional association/a group concerned with children/a group concerned with community betterment,charity or service/any other group.	6 (covering 4 domains: marriage, close friends and relatives, church members hip, group members hip)	People who scored 1 or less on the social network index were compared with those who scored higher; 2 categories: isolated v. not isolated	Not reported	No information re. reliability and validity in this study. Berkman (1977) ackowledged that the extent to which the social network and participation questions measure the relationships and kinds of participation in which respondents are really involved is unknown. Data from Sykes (2002), see below, suggests a degree of validity and reliability.	Once, at baseline	Among people aged 65 to 74, comparing people who scored 1 or less v. scoring higher on the social network index, HR for incidnet stroke: 2.03, 95% CI: 0.96–4.28. Among those aged 75 and over, comparing people who scored 1 or less v. scoring higher on the social network index, HR for incident stroke: 1.36, 95% CI: 0.48–3.81.
Colantonio, 1992	Social network	Berkman-Syme SNI, see above	6 (covering 4 domains: marriage, close friends and relatives, church members hip, group members hip)	The index was scored on a scale from 1 to 12, with the lowest score given to the most isolated subjects; the index scores were dichotomized on the basis of the mean number of networks (x = 3.6)	1,372 subjects (57.2%) had few networks (1-3); 1,178 had larger networks (4+)	No information re. reliability and validity in this study. Berkman (1977) ackowledged that the extent to which the social network and participation questions measure the relationships and kinds of participation in which respondents are really involved is unknown. Data from Sykes (2002), see below, suggests a degree of validity and reliability.	Once, at baseline	When the social network variable was added to a Cox proportional hazards multivariate model looking at incident stroke, X2= 0.15, df=1, p-value=0.6995.

Eng, 2002	Social ties	Berkman-Syme SNI, see above	6 (covering 4 domains: marriage, close friends and relatives, church members hip, group members hip)	The authors reported that 'The index has four levels: low, medium, medium-high, and high (respectively, I, II, III, and IV). Persons with low levels of social ties can be characterized as unmarried, having few friends or relatives, and not being involved in community groups.'	6.0% of the study population was socially isolated (level I of the social network index); 51.3% of subjects were well integrated (level IV).	No information re. reliability and validity in this study. Berkman (1977) ackowledged that the extent to which the social network and participation questions measure the relationships and kinds of participation in which respondents are really involved is unknown. Data from Sykes (2002), see below, suggests a degree of validity and reliability.	Twice, 8 years part - but only the measurement from 1988 was used in the analyses re.disease incidence	Comparing subjects with a low v. high level of social network, total coronary heart disease HR: 0.99, 95% CI: 0.81, 1.20; nonfatal myocardial infarction HR: 1.11, 95% CI: 0.80, 1.53; fatal coronary heart disease HR: 1.82, 95% CI: 1.02, 3.23; sudden cardiac death HR: 0.71, 95% CI: 0.28, 1.81.
Kawachi, 1996	Social network	Berkman-Syme SNI, see above	6 (covering 4 domains: marriage, close friends and relatives, church members hip, group members hip)	Responses to the index were categorised into four levels of social connection: low networks (individuals with low intimate contacts - not married, fewer than 6 friends or relatives - and no membership in either church or community groups), medium networks, medium-high networks and high networks	5.8% of the study population were socially isolated, 23.6% had medium levels of social network, 19.1% had medium-high network levels and 51.5% were sociallyl integrated.	No information re. reliability and validity in this study. Berkman (1977) ackowledged that the extent to which the social network and participation questions measure the relationships and kinds of participation in which respondents are really involved is unknown. Data from Sykes (2002), see below, suggests a degree of validity and reliability.	Once, at baseline	Comparing subjects with a low v. high level of social network, HR for total stroke: 2.02, 95% CI: 1.00, 4.08; HR for fatal stroke (age-adjusted model only): 3.64, 95% CI: 0.78, 16.9; HR for nonfatal stroke: 1.86, 95% CI: 0.85, 4.06; HR for total CHD: 1.14, 95% CI: 0.74, 1.73; HR for fatal CHD: 1.42, 95% CI: 0.72, 2.81; HR for nonfatal CHD: 1.00, 95% CI: 0.58, 1.71; HR for sudden cardiac death: 0.68, 95% CI: 0.16, 2.96; HR for non-sudden cardiac death: 1.89, 95% CI: 0.87, 4.13.

Gafarov, 2013	Social support	Berkman-Syme SNI, see above	6 (covering 4 domains: marriage, close friends and relatives, church membership, group membership)	2 indices: one across all contacts (4 categories: high, average-1, average-2, low) and one re. close contacts only (3 categories: high, average, low).	Across social contacts: 77.7% had low levels of social network; 19.8% had an average social network; 2.5% had high levels of social network. Re. close contacts, 57.1% had low levels of contact, 37.3% had average levels of contact, and 5.7% had high levels of contact.	No information re. reliability and validity in this study. Berkman (1977) ackowledged that the extent to which the social network and participation questions measure the relationships and kinds of participation in which respondents are really involved is unknown. Data from Sykes (2002), see below, suggests a degree of validity and reliability.	Once, at baseline	Comparing subjects with low v. higher levels of social network across the whole sample, HR for myocardial infarction: 2.92, 95% CI 1.040-8.208; HR for stroke: 2.72, 95% CI 1.094-6.763. Comparing subjects with low v. higher levels of social network in the 55-64 age group: HR for myocardial infarction: 5.9, 95% CI: 1.534-22.947. Comparing subjects with low v. higher levels of *close* social network, HR for myocardial infarction :4.9, 95% CI: 1.108-21.762; HR for stroke: 4.1, 95% CI: 1.193-14.055. [unadjusted]
Sykes, 2002	Social support	Berkman-Syme SNI, see above	6 (covering 4 domains: marriage, close friends and relatives, church membership, group membership)	Each item of social support was measured on a scale 0-7, for a final range of 0-28. The score was treated as a continuous predictor variable for analysis.	Not reported	Cronbach's alpha: 0.84. Re. validity, authors reported negative co-variation between social support and hostility (ϕ = - 0.35), and between social support and depression (ϕ = - 0.31).	Once, at baseline	Logistic regression. Hard CHD, full model: X2 = 7.389, df = 5, p-value: not significant. Total angina pectoris, full model: X2 = 16.242, df = 5, p = 0.006. The authors report that the social network variable did not contributed to this effect.

Nagayoshi 2014	Social network	10-item Lubben Social Network Scale: 1. How many relatives do you see or hear from at least once a month? 2. Tell me about the relative with whom you have the most contact: How often do you see or hear from that person? 3. How many relatives do you feel close to? That is how many of them do you feel at ease with, can talk about private matters or can call for help? 4. Do you have any close friends? That is do you have any friends with whom you feel at ease can talk to about private matters or can call on for help? If so how many? 5. How many of these friends do you see or hear from at least once a month? 6. Tell me about the friend with whom you have the most contact. How often do you see or hear from that person? 7. When you have an important decision to make do you have someone you can talk to about it? 8. When other people you know have an important decision to make do they talk to you about it? 9a.Does anybody rely on you to something for them each day? 9b. Do you help anybody with something each day? 10. Do you live alone or with other people?	6 (covering 4 domains: marriage, close friends and relatives, church membership, group membership)	Scores range from 0 to 50 - the higher the score, the larger the social network; 4 categories: score ≤20=small social network; 21 to 25=moderate small social network; 26 to 30=moderate large social network; and ≥31=large social network.'	2.8% subjects had a small social network, 5.7% had a moderately small network, 13.9% a moderately large network, 77.6% a large network	Note that Nagayoshi's study does not focus on people aged 65+, the population for which the Lubben Social Network Scale was developed.	Once, at baseline	Comparing subjects with small v. large network, HR of stroke: 1.44, 95% CI: 1.02–2.04; HR of ischemic stroke: 1.41, 95% CI: 0.98-2.03.
Player, 2007	Social network	10-item Lubben Social Network Scale, see Nagayoshi 2014	10	Scores range from 0 to 50 - the higher the score, the larger the social network; 3 categories: the cohort was divided into tertiles (0 to 35, 36 to 39, 40 to 50.	30.3% had low social networks, 31.2% had moderate networks, 38.5% had high social network levels	Note that Player's study does not focus on people aged 65+, the population for which the Lubben Social Network Scale was developed.	Once, at baseline	χ2 analysis. P-value associated with χ2 in bivariate analysis re. social networks = 0.220

Rosengren 2004	Social integration	Same as Orth-Gomer 1993, see below.	6	Response alternatives were scored from 1 to 6. Scores were divided into quartiles and the variable was categorised into 3 levels of social integration: first quartile: low integration; second and third quartile: moderate integration, last quartile: high integration	The quartile of subjects with lowest scores were classed as having a low level of social integration	According to Unden 1989, internal consistency: Cronbach's alpha = 0.66; split-half reliability: Cronbach's alpha = 0.59.	Once, at baseline	Comparing subjects with high v. low levels of integration, HR for CHD: 0.45, 95% CI: 0.24-0.84.
Orth-Gomer, 1993	Social integration	1. Number of people met during an ordinary week. 2. Number of people with whom respondent shares interests. 3. Number of friends who at any time would come and visit respondent's home and wouldn't be embarrassed if it were untidy. 4. Number of friends or family members with whom respondent can talk frankly. 5. Someone available whom respondent can ask small favors. 6. Someone available—apart from family—to whom respondent can turn in times of difficulties.	6	Responses to items 1-4 were classified into 6 categories, from 0 to more than 15. All other items were coded yes/no and study subjects were divided into quartiles.	157 subjects (21.3%) were in the lower quartile.	According to Unden 1989, internal consistency: Cronbach's alpha = 0.66; split-half reliability: Cronbach's alpha = 0.59.	Once, at baseline	Comparing subjects in the lower v. upper quartiles of social integration, OR for CHD: 3.8, 95% CI: 1.1-13.9.
Kuper, 2006	Social support	1. How many people do you know who share the same interests as you (including people from work and those you meet in your spare time)? 2. How many do you meet and speak with (not counting those who you only meet briefly and/or will not probably meet again)? 3. How many can drop by your home anytime without warning? (For instance, neither you nor they mind if the house is messy, or if you are in the middle of a meal; do not include close relatives). 4. How many can you speak openly with? 5. Apart from your family, can you turn to other people when in trouble? 6. How many people in your surrounding can you ask for favors if necessary?	6	The study population was divided into tertiles based on the responses across all of the women.	15,240 subjects (31.9%) had low social support; 15,807 subjects (33.1%) had intermediary social support; 16,666 subjects (34.9%) had high social support	No information re. validity	Once, at baseline	Comparing subjects with the highest v. lowest social support, HR for myocardial infarction: 1.3, 95% CI: 0.9-1.8.

Ikeda, 2008	Social support	Perceived social support was measured combining questions re.: a) perceived emotional support: “Do you have someone that you can share your intimate feelings and secrets with? (no or yes)”; “Do you have someone that you feel safe and comfortable with? (no or yes)”; “Do you have someone who is supportive of your opinions and actions? (no or yes)”. b) social isolation, defined as not having a friend whom the respondent knew well enough to meet at least once per week: “How many friends do you meet at least once per week: none, 1 to 3 friends, more than 4 friends?”	4	The 4 questions were combined into an overall index of social support. Subjects were categorized into 4 levels based on the distribution of the combined index: low support (scores 0-1), medium (2-3), high (4), very high (5).	10% subjects had low social support, 19% had medium support, 42% had high support, 29% had very high support.	Internal consistency: Cronbach alpha = 0.75.	Once, at baseline	Non-fatal myocardial infarction: comparing subjects with low v. very high levels of support, HR: 0.90, 95% CI: 0.60-1.35; comparing men with low v. very high levels of support, HR: 1.06, 95% CI: 0.68- 1.67; comparing women with low v. very high levels of support, HR: 0.55, 95% CI: 0.19-1.57. Fatal MI: comparing subjects with low v. very high levels of support, HR: 1.00, 95% CI: 0.61-1.63; comparing men with low v. very high levels of support, HR: 1.12, 95% CI: 0.65-1.94; comparing women with low v. very high levels of support, HR: 0.58, 95% CI: 0.17-1.99. Nonfatal stroke: comparing subjects with low v. very high level of support, HR: 1.11, 95% CI: 0.89-1.37; comparing men with low v. very high level of support, HR: 1.09, 95% CI: 0.84-1.43; comparing women with low v. very high level of support, HR: 1.22, 95% CI: 0.85-1.74. Fatal stroke: comparing subjects with low v. very high level of support, HR: 1.45, 95% CI: 1.00-2.10; comparing men with low v. very high level of support, HR: 1.59, 95% CI: 1.01-2.51; comparing women with low v. very high level of support, HR: 1.25, 95% CI: 0.63-2.46.

Reed, 1983	Social network	Two measures were used: 1) One ‘conceptual’ tool combining 9 items re: geographical proximity of parents; of wife’s parents; marital status; number of living children; number of persons in the household; frequency of social activities; frequency of discussing serious problems; frequency of attendance of religious services; number of social organizations attended regularly; 2) and one tool combining the first 5 items listed above (these items were selected based on factor analysis).	9 and 5	Men were divided into quartiles of the two different scores.	Subjects in the lower quartile were classed as having low levels of social network.	Authors acknowledge that the tool has not been validated, justifying their choice based on items used in similar studies.	Once, at baseline	Multiple logistic regression: coefficients associated with social network scores, CHD: beta = -0.0836, p-value > 0.5 nonfatal MI: beta = -0 0576, p-value > 0.5 fatal MI: beta = -0 0505, p-value > 0.5 angina: beta = -0.1348 , p-value > 0.5.
Reed, 1984	Social network	Questionnaire focusing on more intimate ties with relatives and household members: marital status; number of living children; number of persons in the household; geographic closeness of parents.	4	Men were grouped into quartiles based on their social networks summary score.	Subjects in the lower quartile were classed as having low levels of social network.	Authors acknowledge that the tool has not been validated, justifying their choice based on items used in similar studies.	Once, at baseline	Multiple logistic regression analysis. CHD: comparing subjects with low v. high networks, p-value ≤0.05. Stroke: comparing subjects with low v. high networks, p-value >0.05.
Rutledge, 2008	Social network	Social Network Index - The SNI collects information on 12 types of social relationships, including friends, employment, neighbors, marriage partners, belonging to a church, children, parents, in-laws, other relatives, class attendance (e.g., university), volunteer work, and group memberships.	12	Scoring of the SNI produces a measure of social network diversity based on the presence or absence of each of the 12 relationship domains over a 2-week period, with scores ranging from 0 to 12; 2 categories: a dichotomy between high and low scorers was operated based on a cut-off score of 6.	188 (30%) had low social network levels and 441 had high social network levels	No information re. validity or reliability was reported in this study.	Once, at baseline	Comparing subjects with low v. high social network index scores, HR for stroke: 2.7, 95% CI: 1.1-6.5.

Vogt, 1992	Social network - scope, size and frequency	Three indices were created: 1) scope of network, 2) size of network, and 3) frequency of contact within and across network sectors.	15	1) Network scope is the unweighted sum of the number of network domains in which respondents reported one or more relationships. 2) Network size is the combined number of family members, friends, work associates, and neighbors comprising the network. 3) Network frequency is a summed score derived from answers to questions about the frequency with which respondents interacted or participated in activities with others; for each measure, 3 categories: measures were divided into approximate tertiles to the degree that distributions of scores permitted.	Network measures were divided into approximate tertiles to the degree that distributions of scores permitted. For network scope, the mean score was 9.31 (SD = 1.72, range 2-12). The network frequency mean was 13.0 (SD = 3.59, range 0-22). The network size mean was 23.2 (SD = 6.31, range 3-47).	No information reported in this study. Maxwell (1985) reported that the 3 social network indices had low reliability (Cronbach's alpha ranged from .30 for network size to approximately .43 for network scope).	Once, at baseline	Comparing subjects in the low v. high tertiles of social network size: HR for CHD: 1.2, 95% CI: 0.9-1.6; HR for stroke: 0.9, 95% CI: 0.6-1.3, p-value = 0.58.
Barefoot, 2005	Social network /social support	Participants were asked to indicate how frequently they had contact with persons in the following categories: parents, children, other family members, a spouse or partner, colleagues from work (after work), neighbors, and friends. Response options were ‘‘daily,’’ ‘‘weekly,’’ ‘‘monthly,’’ ‘‘rarely,’’ ‘‘never,’’ and ‘‘no one available.’’	7	The responses of ‘‘no one available,’’ ‘‘rarely,’’ and ‘‘never’’ were combined into a ‘‘no contact’’ category. Responses of ‘‘daily,’’ ‘‘weekly,’’ and ‘‘monthly’’ were grouped into one category indicating that contact was present. 2 diversity indices were created by summing responses across multiple classes of contacts. 1) One index was based on all contact sources, with scores ranging from 0 to 7. 2) A second index focused on relationships with parents, children, family, and friends, with scores ranging from 0 to 5. For each measure, there were 6 categories.	Diversity index based on all relationships: mean score on the scale was 3.6 (of a possible score of 7), with 7.5% of participants reporting 1 contact or no contacts and 9.9% reporting 6+ sources of contact. Re. contacts with intimate relationships: The mean score on this index was 2.9 (of a possible score of 5), with 195 (2%) of the participants reporting no contacts and 799 (8%) reporting contacts in all categories.	No data re. reliability or validity were reported in this study.	Once, at baseline	Comparing people with 6-7 contacts to 0-1 contacts, HR for CHD: 0.67, 95% CI: 0.33-1.39. Comparing people with 5 intimate contacts to no contact, HR for CHD: 0.39, 95% CI: 0.14-1.06.

Hedblad, 1992	Contact frequency	Quantitative measure of how often the individual meets with children, kin, neighbours, friends and workmates.	5	A score was calculated by assigning one point to each questionaire item, i.e. minimum score 0, maximum 6 points. 2 categories: total scores wer dichotomised, with low frequency of contact defined as scoring 0-1 point	Not reported.	No data re. reliability or validity were reported in this study.	Once, at baseline	Comparing subjects with low v. high contact frequency, Rrfor CHD: 1.2, 95% CI: 0.3-4.9; regression coefficient = 0.193, SE = 0.713.
**Loneliness and social isolation combined**								
Strodl, 2003	Social support	Duke Social Support Index (DSSI), 11-items. This is a shortened version of the DSSI. The index comprises two subscales: social interaction (i.e., frequency of interactions) and subjective support (i.e., satisfaction with emotional support provided).	11	The scores on the 11 items were combined and categorized as low-fair (score ≤26), high (score 27–29), and very high (score 30–33).	Low to fair social support: 1,579 (17%); high: 3,044; very high: 3,136	Cronbach alpha for this study: 0.62. The authors report that the 11-item DSSI has been validated with an Australian population and found to have reasonable test-retest reliability, concurrent validity, and construct validity (Goodger, Byles, & Higginbotham, 1999).	Once, at baseline	Comparing subjects with low to fair v. very high social support, OR for CHD: 1.41, 95% CI 1.11-1.79. [unadjusted]
Strodl, 2008	Social support	Duke Social Support Index (DSSI), 11-items. This is a shortened version of the DSSI. The index comprises two subscales: social interaction (i.e., frequency of interactions) and subjective support (i.e., satisfaction with emotional support provided). The scores on the 11 items were combined and categorized as low-fair (score ≤26), high (score 27–29), and very high (score 30–33).	11	The scores on the 11 items were combined and categorized as low-fair (score ≤26), high (score 27–29), and very high (score 30–33).	Low-fair social support: 3,613 (41%); high: 3,497; very high: 1,797.	Cronbach alpha for this study: 0.62. The authors report that the 11-item DSSI has been validated with an Australian population and found to have reasonable test-retest reliability, concurrent validity, and construct validity (Goodger, Byles, & Higginbotham, 1999).	Once, at baseline	Comparing subjects with low to fair v. very high social support, OR for stroke: 0.88, 95% CI: 0.62-1.25. [unadjusted]
