# Supplementary material for: Loneliness and social isolation as risk factors for coronary heart disease and stroke: systematic review and meta-analysis of longitudinal observational studies
Source: Heart. 2016 Apr 18;102(13):1009–16. doi: 10.1136/heartjnl-2015-308790 (PMC4941172; doi:10.1136/heartjnl-2015-308790)
Supplement: Supplementary appendix 5 [file heartjnl-2015-308790supp_appendix5.pdf]

Appendix 5 Subgroup analyses of CHD studies

a) According to social relationship domain (loneliness v. social isolation)

Study or Subgroup	log[Risk Ratio]	SE	Weight	Risk Ratio IV, Random, 95% CI
**Loneliness**				
Eaker 1992	1.38629436	0.4161854	25.1%	4.00 [1.77, 9.04]
André-Petersson 2006	0.17395331	0.3173003	32.0%	1.19 [0.64, 2.22]
Thurston 2009	0.42526774	0.1825	43.0%	1.53 [1.07, 2.19]
**Social isolation**				
Gafarov 2013	1.07158362	0.5270221	4.2%	2.92 [1.04, 8.20]
Barefoot 2005	0.39877612	0.3666054	7.6%	1.49 [0.73, 3.06]
Rosengren 2004	0.69314718	0.3511146	8.1%	2.00 [1.00, 3.98]
Ikeda 2008	-0.10536052	0.2069	15.7%	0.90 [0.60, 1.35]
Kuper 2006	-0.26136476	0.1745385	18.3%	0.77 [0.55, 1.08]
Vogt 1992	0.18232156	0.1467793	20.8%	1.20 [0.90, 1.60]
Eng 2002	-0.01005034	0.1002678	25.3%	0.99 [0.81, 1.20]

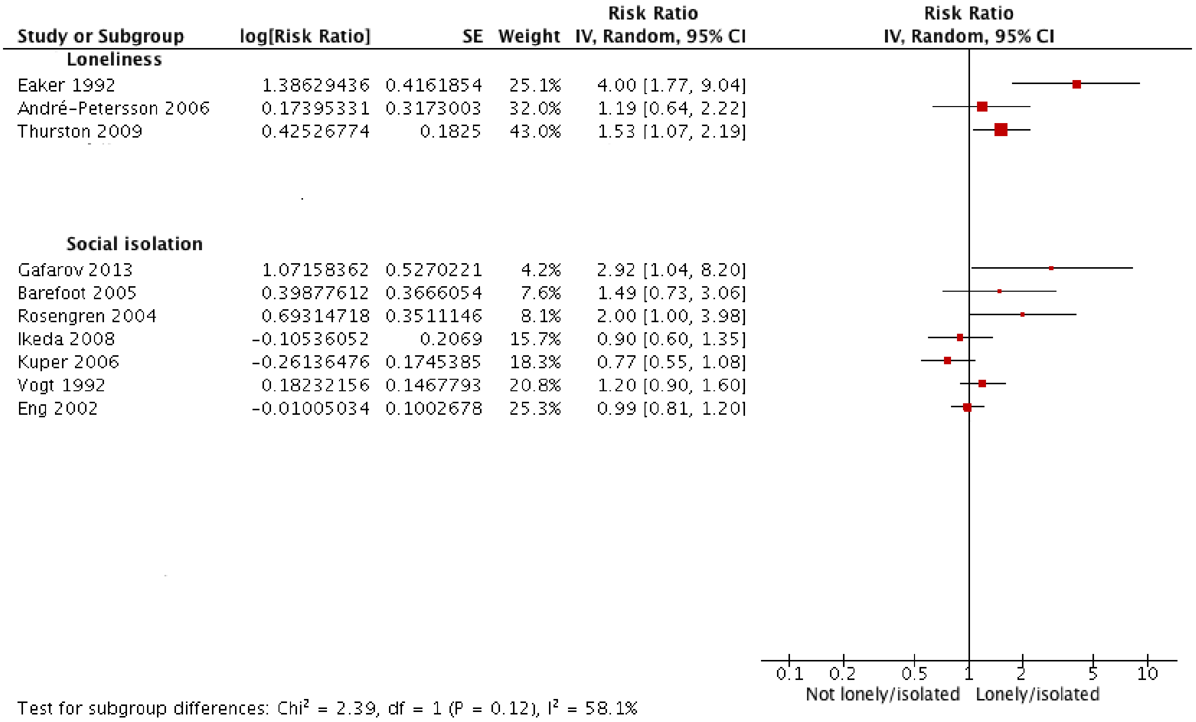

Test for subgroup differences: Chi^2 = 2.39, df = 1 (P = 0.12), I^2 = 58.1%

b) According to gender

Study or Subgroup	log[Risk Ratio]	SE	Weight	Risk Ratio IV, Random, 95% CI
**Men**				
André-Petersson 2006	0.17395331	0.3173003	7.5%	1.19 [0.64, 2.22]
Eng 2002	-0.01005034	0.1002678	66.4%	0.99 [0.81, 1.20]
Ikeda 2008	0.05826891	0.2292099	14.2%	1.06 [0.68, 1.66]
Rosengren 2004	0.69314718	0.3511146	6.1%	2.00 [1.00, 3.98]
Thurston 2009	-0.12783337	0.3624004	5.8%	0.88 [0.43, 1.79]
**Women**				
Eaker 1992	1.38629436	0.4161854	13.8%	4.00 [1.77, 9.04]
Gafarov 2013	1.07158362	0.5270221	10.9%	2.92 [1.04, 8.20]
Ikeda 2008	-0.597837	0.5387361	10.7%	0.55 [0.19, 1.58]
Kuper 2006	-0.26136476	0.1745385	21.6%	0.77 [0.55, 1.08]
Strodl 2003	0.3435897	0.1219042	23.1%	1.41 [1.11, 1.79]
Thurston 2009	0.62593843	0.2285981	19.9%	1.87 [1.19, 2.93]

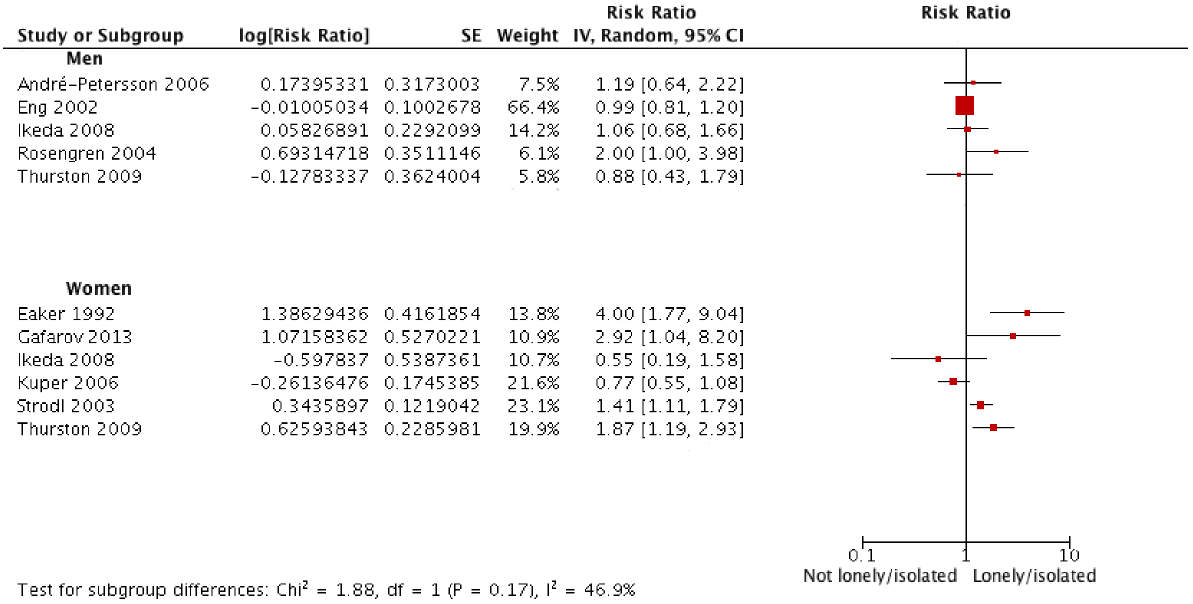

Test for subgroup differences: Chi^2 = 1.88, df = 1 (P = 0.17), I^2 = 46.9%

c) According to risk of confounding

Study or Subgroup	log[Risk Ratio]	SE	Weight	Risk Ratio IV, Random, 95% CI
**Lower risk**				
André-Petersson 2006	0.17395331	0.3173003	9.3%	1.19 [0.64, 2.22]
Barefoot 2005	0.39877612	0.3666054	7.7%	1.49 [0.73, 3.06]
Eaker 1992	1.38629436	0.4161854	6.5%	4.00 [1.77, 9.04]
Eng 2002	−0.01005034	0.1002678	20.6%	0.99 [0.81, 1.20]
Ikeda 2008	−0.10536052	0.2069	14.3%	0.90 [0.60, 1.35]
Rosengren 2004	0.69314718	0.3511146	8.2%	2.00 [1.00, 3.98]
Thurston 2009	0.42526774	0.1825	15.7%	1.53 [1.07, 2.19]
Vogt 1992	0.18232156	0.1467793	17.8%	1.20 [0.90, 1.60]
**Higher risk**				
Gafarov 2013	1.07158362	0.5270221	3.9%	2.92 [1.04, 8.20]
Kuper 2006	0.26136476	0.1745385	33.2%	1.30 [0.92, 1.83]
Strodl 2003	0.3435897	0.1219042	62.9%	1.41 [1.11, 1.79]

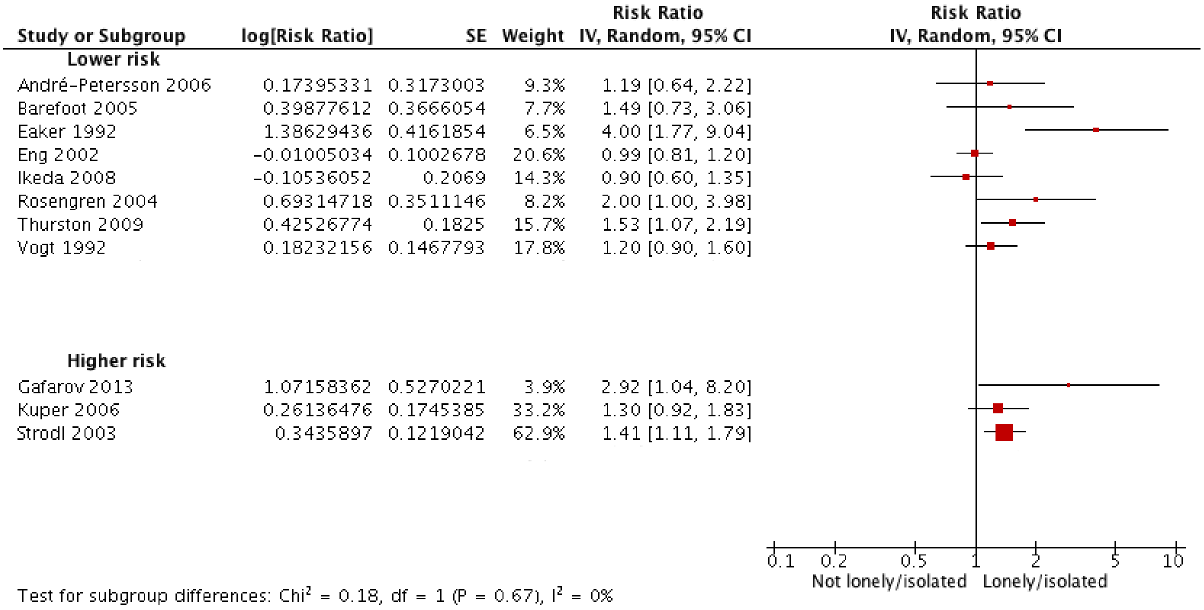

Test for subgroup differences: $Chi^2 = 0.18$, $df = 1$ ($P = 0.67$), $I^2 = 0\%$

d) According to risk of bias due to measurement error – exposure

Study or Subgroup	log[Risk Ratio]	SE	Weight	Risk Ratio IV, Random, 95% CI
**Lower risk**				
Barefoot 2005	0.39877612	0.3666054	6.8%	1.49 [0.73, 3.06]
Eaker 1992	1.38629436	0.4161854	5.7%	4.00 [1.77, 9.04]
Eng 2002	0.01005034	0.1002678	18.3%	1.01 [0.83, 1.23]
Gafarov 2013	1.07158362	0.5270221	3.9%	2.92 [1.04, 8.20]
Ikeda 2008	−0.10536052	0.2069	12.7%	0.90 [0.60, 1.35]
Kuper 2006	0.26136476	0.1745385	14.3%	1.30 [0.92, 1.83]
Rosengren 2004	0.69314718	0.3511146	7.2%	2.00 [1.00, 3.98]
Strodl 2003	0.3435897	0.1219042	17.2%	1.41 [1.11, 1.79]
Thurston 2009	0.42526774	0.1825	13.9%	1.53 [1.07, 2.19]
**Higher risk**				
André-Petersson 2006	0.17395331	0.3173003	17.6%	1.19 [0.64, 2.22]
Vogt 1992	0.18232156	0.1467793	82.4%	1.20 [0.90, 1.60]

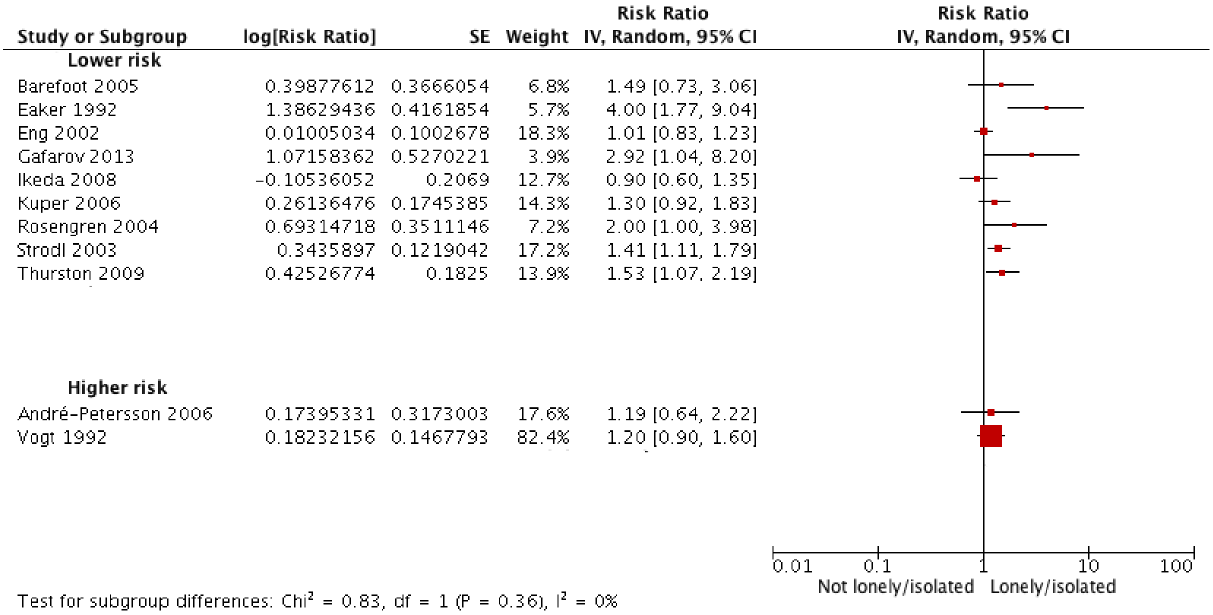

Test for subgroup differences: $Chi^2 = 0.83$, $df = 1$ ($P = 0.36$), $I^2 = 0\%$
