# Supplementary material for: Loneliness and social isolation as risk factors for coronary heart disease and stroke: systematic review and meta-analysis of longitudinal observational studies
Source: Heart. 2016 Apr 18;102(13):1009–16. doi: 10.1136/heartjnl-2015-308790 (PMC4941172; doi:10.1136/heartjnl-2015-308790)
Supplement: Supplementary appendix 6 [file heartjnl-2015-308790supp_appendix6.pdf]

Appendix 6 Subgroup analysis of stroke studies

a) According to risk of confounding

Study or Subgroup	log[Risk Ratio]	SE	Weight	Risk Ratio IV, Random, 95% CI
**Lower risk**				
Avendano 2006 a	0.70803579	0.3813272	7.0%	2.03 [0.96, 4.29]
Avendano 2006 b	0.3074847	0.5284787	3.9%	1.36 [0.48, 3.83]
Ikeda 2008	0.10436002	0.1074	39.9%	1.11 [0.90, 1.37]
Nagayoshi 2014	0.36464311	0.1768265	23.6%	1.44 [1.02, 2.04]
Rutledge 2008	0.99325177	0.4531951	5.1%	2.70 [1.11, 6.56]
Vogt 1992	0.10536052	0.1972459	20.4%	1.11 [0.75, 1.64]
**Higher risk**				
Gafarov 2013	1.00063188	0.464709	26.8%	2.72 [1.09, 6.76]
Kawachi 1996	0.70309751	0.3587	32.1%	2.02 [1.00, 4.08]
Strodl 2008	-0.12783337	0.1788756	41.0%	0.88 [0.62, 1.25]

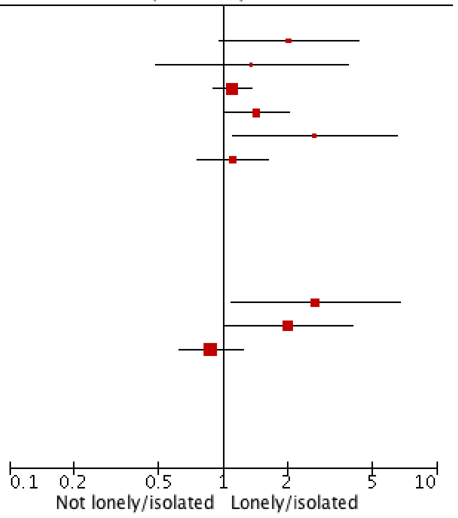

Test for subgroup differences: $Chi^2 = 0.21$, df = 1 (P = 0.65), $I^2 = 0\%$

b) According to risk of bias due to measurement error – outcome

Study or Subgroup	log[Risk Ratio]	SE	Weight	Risk Ratio IV, Random, 95% CI
**Lower risk**				
Gafarov 2013	1.00063188	0.464709	6.0%	2.72 [1.09, 6.76]
Ikeda 2008	0.10436002	0.1074	43.4%	1.11 [0.90, 1.37]
Nagayoshi 2014	0.36464311	0.1768265	27.0%	1.44 [1.02, 2.04]
Vogt 1992	0.10536052	0.1972459	23.6%	1.11 [0.75, 1.64]
**Higher risk**				
Avendano 2006 a	0.70803579	0.3813272	16.7%	2.03 [0.96, 4.29]
Avendano 2006 b	0.3074847	0.5284787	9.7%	1.36 [0.48, 3.83]
Kawachi 1996	0.70309751	0.3587	18.3%	2.02 [1.00, 4.08]
Rutledge 2008	0.99325177	0.4531951	12.6%	2.70 [1.11, 6.56]
Strodl 2003	0.12783337	0.1788756	42.6%	1.14 [0.80, 1.61]

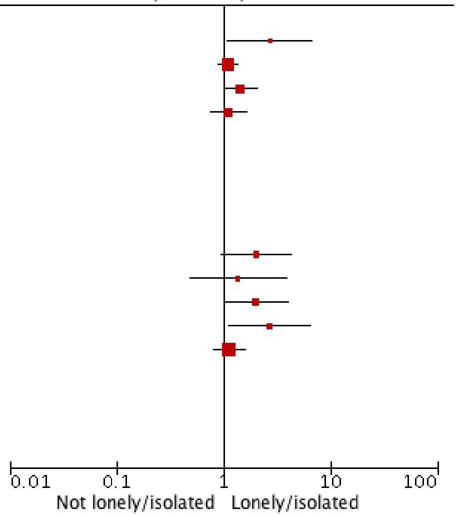

Test for subgroup differences: $Chi^2 = 1.15$, df = 1 (P = 0.28), $I^2 = 13.3\%$
